# Low nanomolar concentrations of Cucurbitacin-I induces G2/M phase arrest and apoptosis by perturbing redox homeostasis in gastric cancer cells *in vitro* and *in vivo*

**DOI:** 10.1038/cddis.2016.13

**Published:** 2016-02-18

**Authors:** C Deng, B Zhang, S Zhang, C Duan, Y Cao, W Kang, H Yan, X Ding, F Zhou, L Wu, G Duan, S Shen, G Xu, W Zhang, M Chen, S Huang, X Zhang, Y Lv, T Ling, L Wang, X Zou

**Affiliations:** 1 Department of Gastroenterology, Drum Tower Hospital, Medical School of Nanjing University, Jiangsu Province, China; 2 Department of Anatomical and Cellular Pathology, State Key Laboratory in Oncology in South China, Prince of Wales Hospital, The Chinese University of Hong Kong, Hong Kong SAR, PR China; 3 Department of Laboratory Medicine, Changhai Hospital, the Second Military Medical University, Shanghai, China; 4 Department of General Surgery, Drum Tower Hospital, Medical School of Nanjing University, Jiangsu Province, China

## Abstract

Cucurbitacin-I (Cu-I, also known as Elatericin B or JSI-124) is developed to inhibit constitutive and abnormal activation of STAT3 in many cancers, demonstrating a potent anticancer activity by targeting disruption of STAT3 function. Here, we for the first time systematically studied the underlying molecular mechanisms of Cu-I-induced gastric cancer cell death both *in vitro* and *in vivo*. In our study, we show that Cu-I markedly inhibits gastric cancer cell growth by inducing G2/M phase cell cycle arrest and apoptosis at low nanomolar concentrations via a STAT3-independent mechanism. Notably, Cu-I significantly decreases intracellular GSH/GSSG ratio by inhibiting NRF2 pathway to break cellular redox homeostasis, and subsequently induces the expression of GADD45*α* in a p53-independent manner, and activates JNK/p38 MAPK signaling. Interestingly, Cu-I-induced GADD45*α* and JNK/p38 MAPK signaling form a positive feedback loop and can be reciprocally regulated by each other. Therefore, the present study provides new insights into the mechanisms of antitumor effects of Cu-I, supporting Cu-I as an attractive therapeutic drug in gastric cancer by modulating the redox balance.

Despite a steady decline in gastric cancer incidence and mortality in the majority of developed countries, it is currently one of the most common malignancies and a leading cause of cancer-related death worldwide, particularly in some countries with historically high incidence (China, Japan, and Korea).^1, 2^ Most gastric cancer patients are diagnosed at advanced stages and thus may no longer be candidates for curative therapies. Chemotherapy using a number of different combinations of agents (that is, 5-Fluorouracil, Adriamycin, Cisplatin, and so on) has been the common treatment for gastric cancers patients. However, they provide limited benefits for patients at advanced stages due to the low response rate and high rate of multidrug resistance.^3^ Thus, there is clearly an urgent need to develop more efficacious therapeutics to treat advanced gastric cancers.

Cucurbitacin-I (Cu-I), also known as Elatericin B or JSI-124, was originally identified to be a potent selective inhibitor of the Janus kinase 2/signal transducer and activator of transcription 3 (JAK2/STAT3) signaling pathway with antiproliferative and antitumor properties.^4, 5, 6, 7^ Upon inhibition of STAT3-dependent gene transcription, Cu-I elicits antiproliferative effects in breast, glioma, head and neck squamous carcinoma, and lung cancer cells with activated STAT3 signaling.^4, 6, 8, 9^ However, the anticancer effect and underlying mechanism of Cu-I in human gastric cancer is still elusive.

In the present study, we show that Cu-I markedly inhibits the growth of gastric cancer cell lines by inducing G2/M phase cell cycle arrest and apoptosis at low nanomolar concentrations. Interestingly, mechanistic analysis revealed that the effect of Cu-I is independent of STAT3 signaling but rather involves the disruption of the balance between pro-oxidants (ROS generation) and antioxidants (mainly expressed by the GSH/GSSG ratio). Furthermore, to the best of our knowledge, we revealed for the first time that Cu-I could efficiently inhibit NRF2 and its downstream targets, whose main function is to modulate GSH generation.^10^ Finally, we confirmed our *in vitro* observations by showing profound antitumor activity of Cu-I in a xenograft model with no apparent toxicity to the mice. Our findings collectively indicated that Cu-I may become a potential therapeutic agent against human gastric cancer in the future.

## Results

### Low nanomolar concentrations of Cu-I inhibits viability of human gastric cancer cells independent of its anti-STAT3 activity

**Figure 1:**
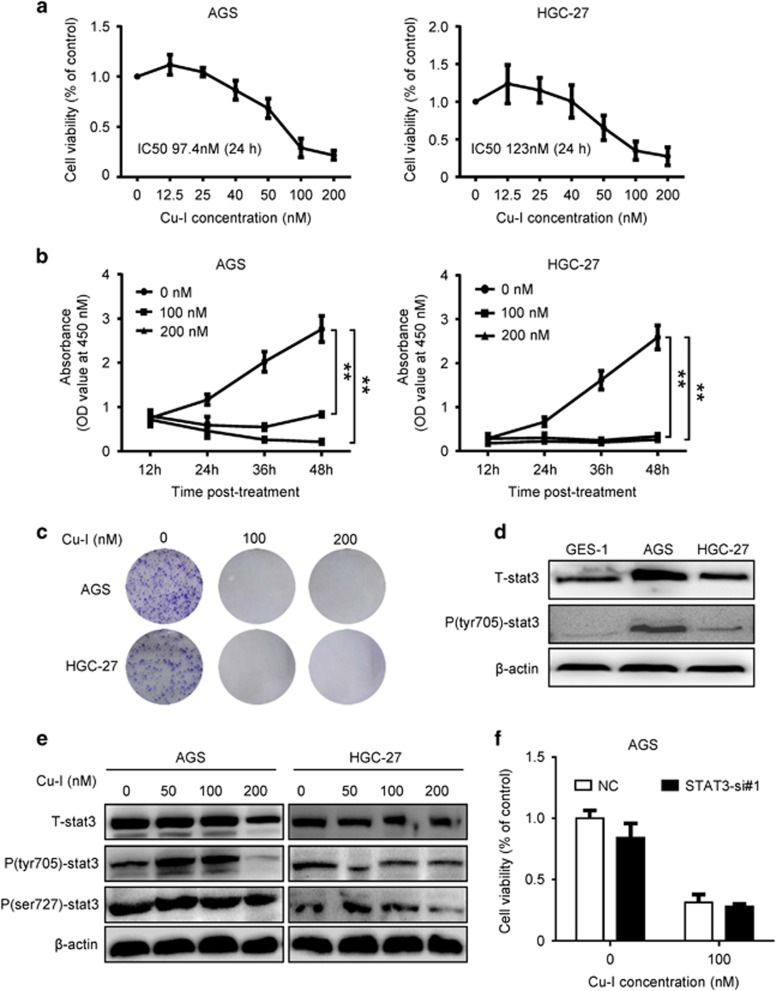
Cu-I inhibits viability of human gastric cancer cells independent of its anti-STAT3 activity at low nanomolar concentrations. (**a**) AGS and HGC-27 cells were treated with vehicle (0.1% DMSO) or varying concentrations of Cu-I for 24 h and assayed by CCK-8. Cell viability was calculated by the following formula: relative cell viability=(absorbance_450nm_ of treated group−absorbance_450nm_ of blank)/(absorbance_450nm_ of control group−absorbance_450nm_ of blank). (**b**) AGS and HGC-27 cells were treated with 100 nM and 200 nM of Cu-I over a course of 48 h, relative absorbance at 450 nM was analyzed to represent as time-dependent antitumor effect of Cu-I. Each data represent mean±S.D. of three independent experiments done in triplicates. *P<0.05, **P<0.01. (**c**) Representative experiment of colony-formation assay of Cu-I-treated AGS and HGC-27 cells. Both cells were grown for 14 days treated with 100 nM and 200 nM of Cu-I, and stained with 0.5% crystal violet. (**d**) The protein levels of total and phosphorylated forms of STAT3 were analyzed by western blotting in GES-1 and AGS and HGC-27 cell lines. (**e**) Western blot analysis of total and phosphorylated STAT3 in AGS and HGC-27 cells untreated or treated with increasing concentration of Cu-I, respectively. (**f**) AGS cells were transfected with either negative control siRNA or STAT3-si#1 for 48 h, and then followed by 100 nM Cu-I for another 24 h. The cell viability was determined by using CCK-8

AGS and HGC-27 cells were incubated in medium with Cu-I for 24 h over a range of concentrations (0, 12.5, 25, 40, 50, 100, and 200 nM). And the cell viability was evaluated by CCK-8 assay. Cu-I treatment inhibited proliferation of both cell lines in a dose-dependent manner. The IC_50_ values of Cu-I, which were ~97.4 nM and 123 nM in AGS and HGC-27 cell lines, respectively, were much lower than those reported in other type of cancer cells (Figure 1a).^4, 7^ We next treated both cell lines with 100 nM and 200 nM of Cu-I over a course of 48 h. The CCK-8 assay showed that Cu-I treatment generated a maximal inhibition of cell viability rapidly (as soon as 24 h, Figure 1b). Furthermore, Cu-I treatment almost completely inhibited the formation of AGS and HGC-27 cell colonies as determined by colony-formation assays (Figure 1c,Supplementary Figure 1a and 1b). Taken together, these data support a suppressive role for Cu-I, which might inhibit gastric cancer cell growth at low nanomolar concentrations.

A number of lines of evidence suggested the antitumorigenic activity of Cu-I is mainly due to their propensity to inhibit JAK2/STAT3 signaling,^4, 5, 6, 7^an important oncogenic pathway constitutively activated in a number of cancers.^11, 12, 13^ Although both gastric cancer cell lines had constitutive activation of STAT3 compared with the human normal gastric epithelial cell line GES-1 (Figure 1d), there was no obvious inhibition of Tyr705 or Ser727 phosphorylation of STAT3 in both cell lines following Cu-I treatment at concentrations of 100 nM, which is close to the value of IC_50_ and inhibits cell growth effectively, whereas a notable reduction of Tyr705 phosphorylation in AGS cells and a marginal Ser727 phosphorylation in HGC-27 cells treated with 200 nM of Cu-I, respectively, were observed (Figure 1e), suggesting that phosphorylation of STAT3 may be dispensable for the inhibitory role of Cu-I. To further demonstrate that STAT3 pathway is not involved in cell growth inhibtion by Cu-I treatment at low concentrations, we used three individual siRNAs to specifically knock down the endogenous expression of STAT3 in AGS cells (Supplementary Figure 1c), however, gene silencing of STAT3 failed to restore the Cu-I-inhibited cell growth (Figure 1f). Collectively, these data indicate that the antitumorigenic activity of Cu-I was independent of its anti-STAT3 activity in gastric cancer cell lines.

### Cu-I leads to G2/M cell cycle arrest and apoptosis in gastric cancer cells via induction of GADD45*α*

**Figure 2:**
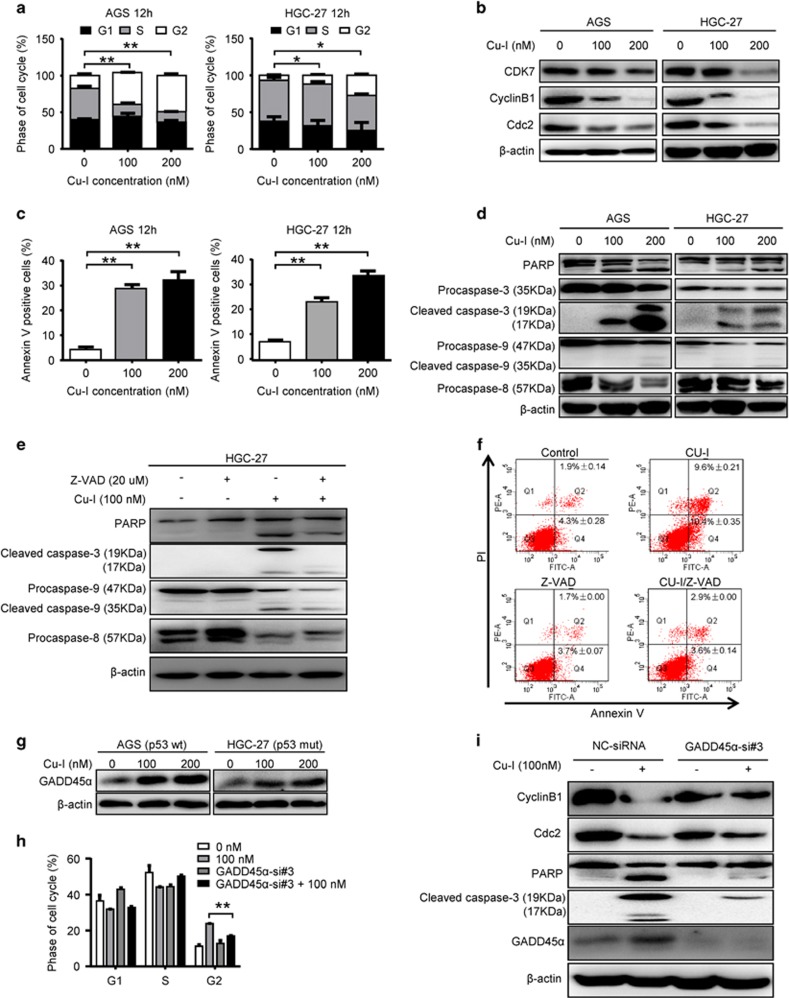
Cu-I leads to G2/M cell cycle arrest and apoptosis in gastric cancer cells via induction of GADD45*α*. (**a**) Cell cycle analysis of AGS and HGC-27 cells after treatment with 0, 100, 200 nM Cu-I for 12 h. *P<0.05, **P<0.01. (**b**) The protein expression of CDK7, cyclin B1, and CDC2 in AGS and HGC-27 cells treated with 0, 100, 200 nM Cu-I for 24 h was evaluated by western blotting. (**c**) Annexin V levels of AGS and HGC-27 cells after treatment with 0, 100, 200 nM Cu-I for 12 h were assayed by flow cytometry. **P<0.01. (**d**) Cleavage of caspase-9, caspase-8, caspase-3, and PARP was evaluated by western blotting in both cell lines after treatment with Cu-I for 24 h. (**e**) HGC-27 cells were treated with 100 nM Cu-I with or without pan-caspase inhibitor (Z-VAD-FMK, 20 *µ*M) for 24 h, then whole-cell lysates were separated by SDS-PAGE and reacted with indicated antibodies. (**f**) HGC-27 cells were treated as described above for 12 h, Annexin V/PI double staining was assayed by flow cytometry. (**g**) Western blot analysis of Cu-I-induced GADD45*α* protein expression in AGS and HGC-27 cells. (**h**) AGS cells were transfected with either negative control siRNA or GADD45*α*-si#3 for 48 h, and then followed by treatment of 100 nM of Cu-I for 12 h, the percentage of phase of cell cycle was determined by flow cytometry. **P<0.01, compared with Cu-I alone-treated group. (**i**) AGS cells were transfected with either negative control siRNA or GADD45*α*-si#3 for 48 h, and then followed by treatment of 100 nM of Cu-I for 24 h; whole-cell lysates were separated by SDS-PAGE and then reacted with indicated antibodies

To further study the inhibitory effect of Cu-I on cell growth, we investigated the cell cycle distribution in both cell lines by flow cytometry. Cu-I treatment for 12 h significantly induced G2/M phase arrest in both cell lines (Figure 2a and Supplementary Figure 1d). Concordantly, the expression of CDK7, cyclin B1, and CDC2, which are known to regulate G2/M phase transition, were markedly reduced (Figure 2b). After ~1 h exposure to Cu-I at 100 nM, both cell lines became shrunken, round, detached, or even floated, suggesting the apoptotic events may be present (Supplementary Figure 1e). Thus, we used Annexin V/PI assay to evaluate whether Cu-I could induce apoptosis in gastric cancer cells. Treatment of both cell lines with Cu-I for 12 h markedly increased apoptosis (Figure 2c and Supplementary Figure 1 f). Consistently, caspase-9, caspase-8, and caspase-3 as well as PARP cleavage were activated by Cu-I in a dose-dependent manner (Figure 2d). To further demonstrate the requirement of caspase activity for cell death induced by Cu-I, HGC-27 cells were treated with 100 nM Cu-I in the absence or presence of pan-caspase inhibitor (Z-VAD-FMK, 20* µ*M). As shown in Figure 2e, the activation of caspase-9, caspase-8, and caspase-3 as well as PARP cleavage trigged by Cu-I was partially abrogated by Z-VAD-FMK. Subsequently, Annexin V/PI double staining demonstrated that pretreatment with Z-VAD-FMK apparently inhibited the apoptotic cell death induced by Cu-I (Figure 2f). These data add further evidence for the inhibitory role of Cu-I against gastric cancer.

GADD45*α* has a pivotal role in cellular stress responses and is implicated in DNA damage repair, G2/M cell cycle arrest, and apoptosis.^14, 15^ GADD45*α* mRNA is downregulated in gastric cancer (Supplementary Figure 2a). Moreover, elevated GADD45*α* mRNA is associated with a better prognosis and improved survival for gastric cancer patients (Supplementary Figure 2b). These data suggest GADD45*α* may be a tumor suppressor gene in gastric cancer. Interestingly, Cu-I treatment induced GADD45*α* protein markedly in both cell lines (Figure 2g). We then determined whether Cu-I-G2/M arrest and apoptosis was mediated by GADD45*α*. By using siRNA-targeting GADD45*α* mRNA (Supplementary Figure 2c), we found GADD45*α* depletion partly attenuated G2/M arrest induced by Cu-I and reversed Cu-I-inhibited cyclin B1 and CDC2 expression in AGS cells (Figures 2h and i). In addition, depletion of GADD45*α* partially attenuated Cu-I-induced caspase-3 and PARP cleavage (Figure 2i), suggesting the apoptosis was also compromised. Altogether, our data indicate that Cu-I leads to G2/M cell cycle arrest and apoptosis in gastric cancer cells at least partially via induction of GADD45*α*.

### Cu-I induces DNA damage and GADD45*α* expression through a p53-independent manner

**Figure 3:**
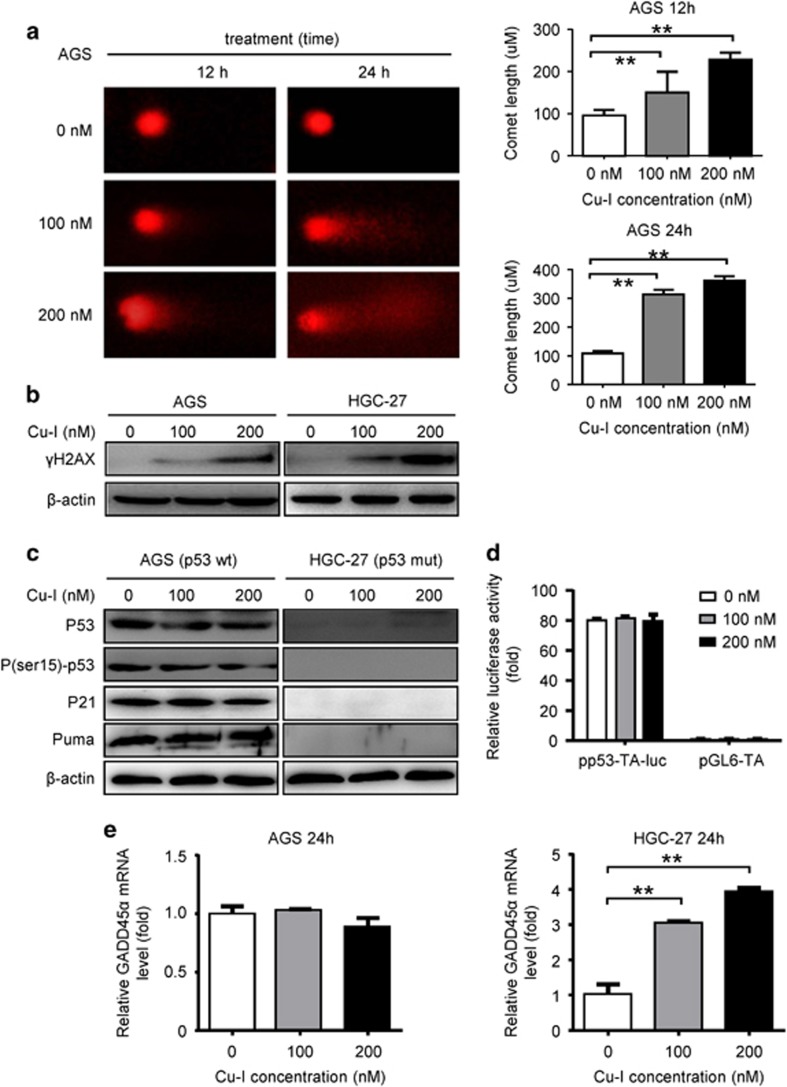
Cu-I induces DNA damage and GADD45*α* expression through a p53-independent manner. (**a**) Typical SCGE pictures (left) of the control group, 100 nM and 200 nM treatment group for 12 h and 24 h. Measurements were conducted in triplicate. (× 400). (right) Quantitative analysis of comet tail lengths from the SCGE pictures. **P<0.01. (**b**) Western blotting analysis of the expression levels of *γ*H2AX. (**c**) Western blotting analysis of the expression levels of p53 itself, P(ser15)- p53 and its other transcriptional targets (p21 and puma) in both AGS and HGC-27 cells. (**d**) P53 transcription activity in Cu-I-treated AGS cells was evaluated by using p53 response element luciferase activity assay. (**e**) GADD45*α* mRNA level in AGS and HGC-27 cells treated by Cu-I for 24 h was assayed by quantitative real-time PCR. *β*-actin served as an internal control. **P<0.01

Apoptotic cell death is always triggered by DNA damage via p53-dependent or -independent manners.^16^ Comet assay was thus conducted to determine whether Cu-I induced DNA damage in gastric cancer cells. After 12 h treatment, the comet tail lengths were 150.0±35.0 *μ*m and 228.0±12.0 *μ*m, respectively, which was longer than that of control group. After 24 h treatment, the comet tail lengths increased to 313.5±11.5 *μ*m and 361.0±11.0 *μ*m, respectively, which were much longer than that of the control cells (Figure 3a). Meanwhile, increased of *γ*H2AX, an early sign of DNA damage,^17^ was also observed in both AGS (p53-wild type) and HGC-27 (p53-mutated) cell lines upon Cu-I treatment (Figure 3b). These results collectively indicated that Cu-I treatment could induce DNA damage in gastric cancer cells, regardless of their p53 status via a dose- and time-dependent manner.

GADD45*α* is a well-known p53-responsive stress protein. Our data, however, excluded the role of p53 in inducing GADD45*α* in gastric cancer cells following Cu-I treatment. First, GADD45*α* protein is induced by Cu-I in both AGS (p53-wild type) and HGC-27 (p53-mutated) cell lines (Figure 2g). Second, Cu-I treatment does not activate p53 signaling because p53 itself, phosphorylated-p53, and its transcriptional targets (that is, p21 and puma) were not induced in AGS cells (Figure 3c). The luciferase activity of a reporter plasmid harboring a wild-type p53 response element showed virtually no responsiveness to treatment of AGS cells with Cu-I (Figure 3d). Third, GADD45*α* mRNA is induced in HGC-27 cells instead of AGS cells following Cu-I treatment (Figure 3e). These data suggest that GADD45*α* might be induced by Cu-I through a p53-independent manner.

### Cu-I induces JNK/p38 MAPK activation in gastric cancer cells

**Figure 4:**
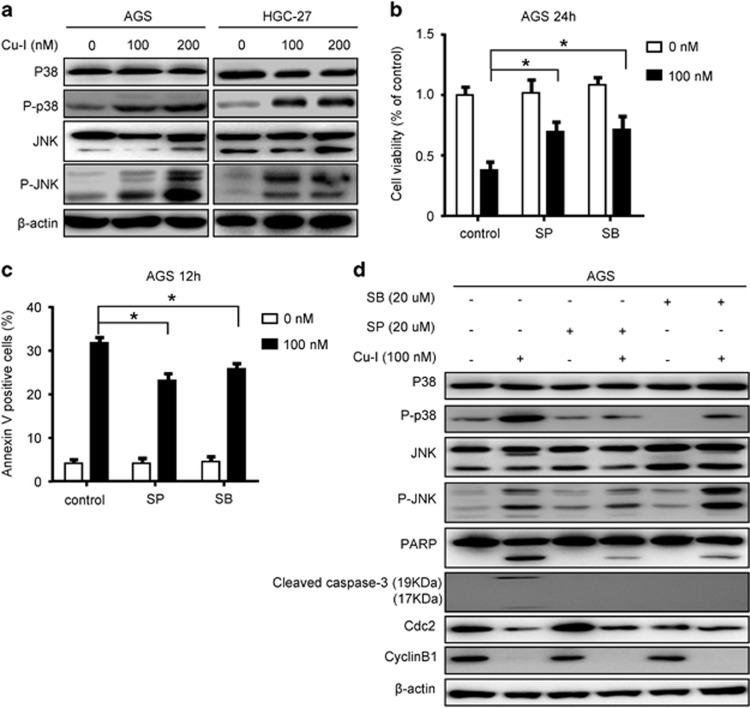
Cu-I induces JNK/p38 MAPK activation in gastric cancer cells. (**a**) Western blotting analysis of total and phosphorylated p38 and JNK in AGS and HGC-27 cells after treatment with 0–200 nM of Cu-I for 24 h. (**b**–**d**) AGS cells were pretreated with the JNK inhibitor (SP600125, 20 *µ*M) or p38 MAPK inhibitor (SB203580, 20* µ*M) for 1 h, and then treated with 100 nM Cu-I. (**b**) After treatment for 24 h, the cell viability was assayed by CCK-8. (**c**) After treatment for 12 h, the percentage of apoptosis in treated AGS cells was assayed. Each data were shown as mean±S.D. of at least three independent experiments done in triplicates. *P<0.05, compared with Cu-I alone-treated group. (**d**) After treatment for 24 h, western blot analysis of cell lysates was performed using indicated antibodies

It has been reported that activation of MAPK subfamilies by cucurbitacins could induce oxidative stress-mediated cell death.^18^ To evaluate whether MAPKs activation was involved in inhibition of cell proliferation in gastric cancer cells treated with Cu-I, we examined the changes of two major MAPK pathways in both cell lines exposed to Cu-I for 24 h. There was a considerable JNK and p38 activation as evidenced by the increased phosphorylation of JNK and p38 proteins in both cell lines (Figure 4a). Pretreatment of AGS cells with SP600125 (JNK inhibitor) or SB203580 (p38 inhibitor) partly reversed Cu-I-inhibited cell viability (Figure 4b) by reducing the resultant apoptosis (Figure 4c). Consistently, caspase-3/PARP activation was also partly inhibited in cells pretreated with JNK or p38 inhibitor. However, there was no evident change in expression of the cell cycle regulators cyclin B1 and CDC2 (Figure 4d). This result suggests that JNK and p38 activation in gastric cancer cells following Cu-I treatment inhibits cell viability mainly by inducing apoptotic cell death.

### GADD45*α* forms a positive feedback loop with JNK/p38 MAPK to mediate gastric cancer cell apoptosis and is driven by decreased intracellular GSH/GSSG ratio regulated by NRF2 pathway

**Figure 5:**
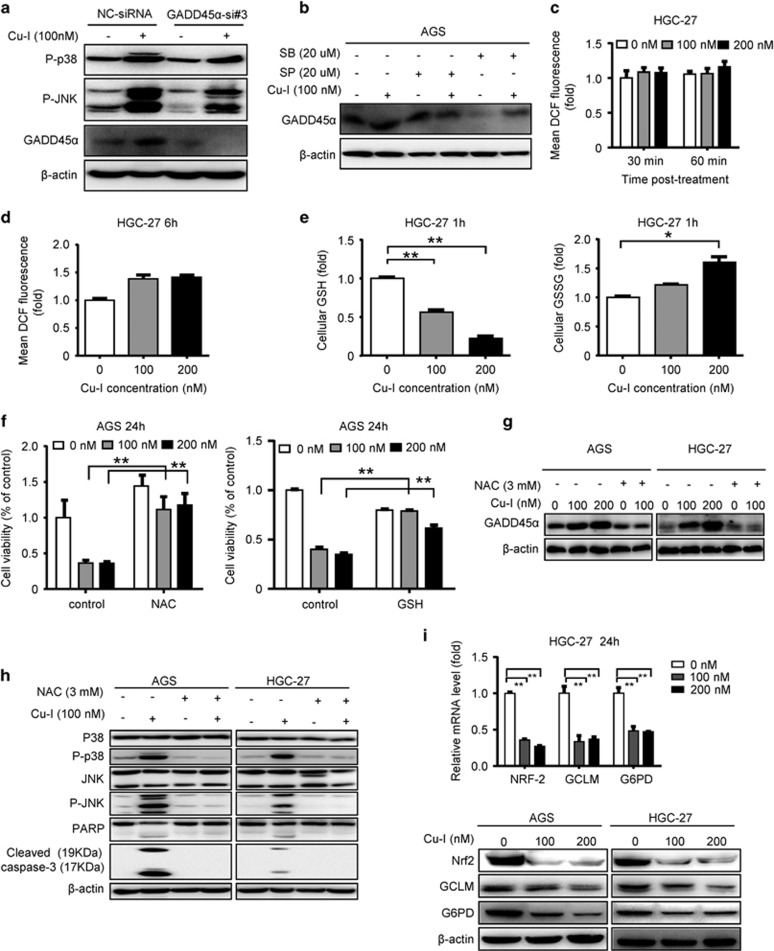
GADD45*α* forms a positive feedback loop with JNK/p38 MAPK to mediate gastric cancer cell apoptosis and is driven by decreased intracellular GSH/GSSG ratio regulated by NRF2 pathway. (**a**) AGS cells were transfected as described in Figure 2i, and then treated with 100 nM Cu-I for 24 h, cell lysates were collected for western blot analysis with indicated antibodies. (**b**) AGS cells were treated as described in Figure 4d. The expression levels of GADD45*α* protein were measured by western blotting. (**c**, **d**) HGC-27 cells were treated by Cu-I (0–200 nM) for various times, as indicated. The redox-sensitive probe 2′-7′-dichlorofluorescein diacetate (DCF-DA, 5 *µ*M) was incubated for 30 min after treatment. Fluorescence intensity was measured immediately after incubation by fluorescence microplate assay in 96-well plates (**c**) or by flow cytometry analysis (**d**). Bars represent S.D. from three independent experiments performed in triplicates. (**e**) GSH and GSSG levels were evaluated in HGC-27 cells treated by Cu-I for 1 h. Bars represent S.D. of three independent experiments performed in triplicates. *P<0.05, **P<0.01. (**f**) After pretreatment with 3 mM NAC or 4 mM GSH for 1 h, AGS cells were then incubated with indicated concentrations of Cu-I for 24 h. Cell viability was determined by CCK-8. **P<0.01, compared with Cu-I alone-treated group. (**g**, **h**) AGS and HGC-27 were pretreated with 3 mM NAC, then treated with indicated concentrations of Cu-I for 24 h. Protein levels of GADD45*α* (**g**) and JNK/p38 as well as subsequent caspase-3/PARP activation were verified by western blotting (**h**). (**i**) NRF2, GCLM, and G6PD mRNA levels in HGC-27 cells (top) treated by Cu-I for 24 h were assayed by real-time PCR. *β*-actin served as an internal control. **P<0.01. (bottom) the protein expression levels of NRF2, GCLM, and G6PD in AGS and HGC-27 cells treated by Cu-I for 24 h

Previous report showed that GADD45*α* elicits its function through activation of the stress-induced JNK and p38 kinases, which contribute to increase in apoptosis and senescence.^15^ Consistently, in AGS cells where GADD45*α* gene was silenced by using siRNA, p38 and JNK activation induced by Cu-I was efficiently inhibited (Figure 5a). This observation, in combination with the partially attenuated caspase-3 and PARP cleavage (Figure 2i), suggests that the tumor-suppressive function of GADD45*α* is partly mediated by JNK and p38 activation, which in turn resulted in apoptosis of gastric cancer cells. Conversely, both JNK and p38 MAPK signaling may positively regulate GADD45*α* in various cell types.^19, 20^ In agreement with the findings in other cells, pretreatment of gastric cancer cells with JNK inhibitor abolished the resultant induction of GADD45*α* by Cu-I, and pretreatment with p38 inhibitor notably decreased the GADD45*α* expression, although its induction by Cu-I still existed (Figure 5b). These findings, together with the compromised apoptosis led by respective inhibitor (Figures 4c and d), indicate that JNK and p38 MAPK signaling was involved in the induction of GADD45*α* and apoptosis by Cu-I in gastric cancer cells. We then speculated that GADD45*α* acts in a positive feedback loop with JNK/p38 MAPK to mediate gastric cancer cell apoptosis led by Cu-I.

Both GADD45*α* and MAPK can be modulated by reactive oxygen species (ROS) formation,^21, 22^ which can be induced by Cu-I.^18, 23^ To decipher whether ROS was involved in the antitumor activities of Cu-I, we assessed ROS levels by determining the mean fluorescence intensity of dichlorofluorescein (DCF) with the redox-sensitive probe 2'-7'-dichlorofluorescein diacetate (DCF-DA) in HGC-27 cells after exposure to Cu-I for 30 and 60 min, respectively. There was no significant increase of ROS in cells treated with Cu-I (either 100 nM or 200 nM) (Figure 5c). We failed to find a marked increase of ROS accumulation in HGC-27 or AGS cells even treated by Cu-I for 6 h (Figure 5d and Supplementary Figure 3a). Mitochondria are considered to be a major source of ROS. To further rule out the involvement of ROS, MitoSOX-based measurement using flow cytometry was employed to measure superoxide production in the mitochondrial matrix. The data revealed no significant increase of mitochondrial superoxide in AGS or HGC-27 cells treated by Cu-I for 6 h (Supplementary Figure 3b). There is a balance between ROS and antioxidant systems, which is essential for cell survival. Cellular antioxidant systems are mainly controlled by GSH system that scavenges intracellular ROS to prevent cell death induced by stress or drugs.^24^ We then determined whether Cu-I could change the redox status of cells. Levels of GSH and GSSG in HGC-27 cells following treatment with Cu-I for 1 h were measured. As shown in Figure 5e, Cu-I remarkably reduced GSH levels but increased GSSG levels in HGC-27 cells in a dose-dependent manner. The intracellular GSH/GSSG ratio, an indicator of overall redox environment of cells, was consequently decreased (Supplementary Figure 3c). Co-treatment with *N*-acetyl-l-cysteine (NAC), a substrate for synthesis of GSH,^25^ or exogenous GSH, nearly completely eliminated Cu-I-induced growth inhibition in both cell lines (Figure 5f and Supplementary Figure 3d). Concordantly, colony-formation assay showed the difference of the colony size and number between AGS cells untreated and treated by Cu-I was nearly abolished by co-treatment with NAC (Supplementary Figure 3e). Moreover, the induction of GADD45*α* by Cu-I was completely blocked by NAC in both cell lines (Figure 5g). Similarly, the activation of JNK/p38 MAPK signaling and subsequent caspase-3/PARP cleavage was also repressed by NAC (Figure 5h). Altogether, these results suggest that the inhibitory effect of Cu-I may be originally attributed to its capacity in modulating intracellular GSH/GSSG ratio instead of markedly increasing ROS.

To further explore the specific molecular target of Cu-I in decreasing intracellular GSH/GSSG ratio, we analyzed the change of NRF2 pathway after Cu-I treatment in gastric cancer cells. NRF2 is the sole controller of the enzymes responsible for producing GSH. Targets of NRF2 such as glucose-6-phosphate dehydrogenase (G6PD) and glutamate–cysteine ligase complex modifier subunit (GCLM) have prominent roles in promoting cancer cell survival by neutralizing the toxic effects of oxidative stress. GCLM is involved in the catalytic reaction of glutamate with cysteine, which is the rate-limiting step in GSH synthesis. G6PD is responsible for NADPH production, leading to subsequent GSH regeneration.^10^ Interestingly, we found that Cu-I inhibits the expression of NRF2 at both mRNA and protein levels. In addition, the downstream targets responsible for GSH generation and regeneration, GCLM and G6PD, were also suppressed (Figure 5i and Supplementary Figure 3 f). Therefore, these data indicated that Cu-I might be a potential inhibitor of NRF2 pathway to break the redox homeostasis of the gastric cancer cells.

### Cu-I inhibits *in vivo* growth of subcutaneous xenograft tumors of human gastric cancer cells

**Figure 6:**
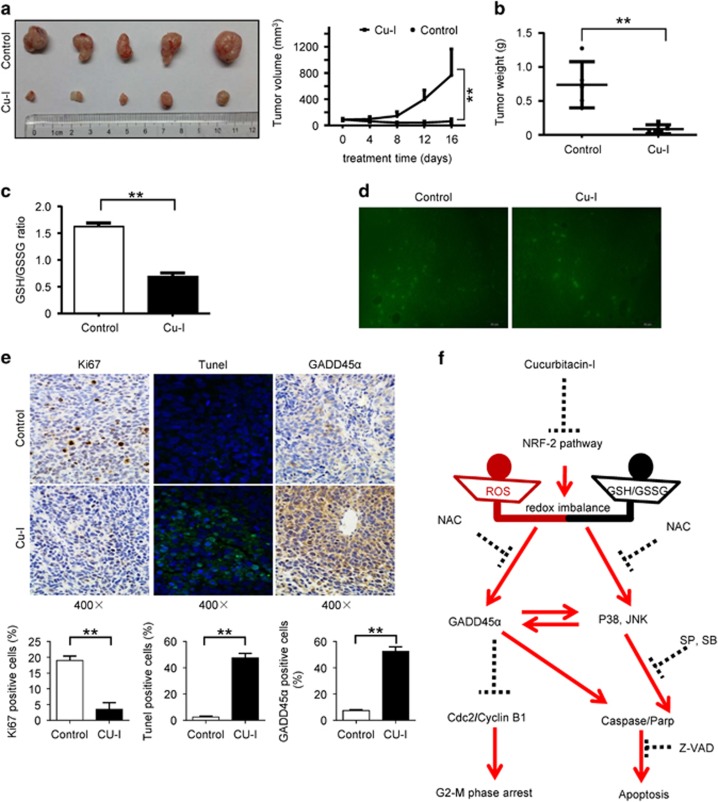
Cu-I inhibits *in vivo* growth of subcutaneous xenograft tumors of human gastric cancer cells. (**a**) Left, resected tumors from each group (*n*=5) were photographed. Right, increase of tumor volumes was plotted. (**b**) Tumor weight in each group was recorded. **P<0.01. (**c**) Tumor lysates analysis of ratio of GSH/GSSG in each group. (**d**) Representative micrographs showing DCF fluorescence in resected tumors. (**e**) Representative immunohistochemistry, immunofluorescence micrographs, and quantitative data displayed as mean±S.D. showing ki67 (right), TUNEL (middle), and GADD45*α* (left) staining, respectively (× 400). (**f**) Schematic illustration of possible molecular mechanism of Cu-I-mediated cell growth inhibition in gastric cancer

We further investigated the effects of Cu-I in gastric cancer cells *in vivo*. HGC-27 cells were subcutaneously implanted in nude mice, and the experimental mice were given 1 mg/kg of Cu-I intraperitoneally daily. Growth of the tumors was significantly inhibited in the mice treated by Cu-I compared with that in control mice (Figure 6a). The overall tumor weight was also significantly reduced in the mice that received Cu-I (Figure 6b). Consistent with the result obtained in cancer cell lines *in vitro*, the intracellular GSH levels (Supplementary Figure 4a), and GSH/GSSG ratio (Figure 6c) in Cu-I-treated tumors were substantially reduced relative to the control tumors, whereas the overall GSSG levels were similar between Cu-I-treated and control tumors (Supplementary Figure 4b). Cu-I failed to induce a significant increase of ROS production in Cu-I-treated tumors (Figure 6d). Furthermore, Cu-I-treated tumors displayed significantly less-proliferative cells as determined by Ki-67 staining, and increased cell apoptosis as determined by TUNEL assay (Figure 6e, left, and middle). Moreover, immunohistochemical staining confirmed increase of GADD45*α* in Cu-I-treated tumors (Figure 6e, right). Finally, treatment with Cu-I was without apparent ill consequences for the mice such as altered weight (Supplementary Figure 4c), toxicity in the lung, liver, spleen and kidney (Supplementary Figure 4d), signs of discomfort, or impaired movement (data not shown). These data strongly support Cu-I as a promising therapeutic agent against human gastric cancer without apparent side-effects.

## Discussion

Cellular redox homeostasis was identified to have a central role in a multitude of physiological and pathophysiological processes.^26^ Redox balance is achieved by various enzyme systems that neutralize toxic oxidants, such as ROS, which causes dysfunction of antioxidant defense mechanism in gastric mucosal, leading to DNA damage, accelerating cell death including apoptosis and subsequent cell proliferation, and therefore leading to the pathogenesis of gastric disorders as well as carcinogenesis.^27^ On the other hand, GSH has indispensable roles in cellular redox homeostasis by promoting ROS clearance, whereas GSH deficiency leads to oxidative stress.^28^ Recently, extensive work has been conducted and led to a multitude of new anticancer therapeutic agents, which increase the levels of ROS and/or suppress the antioxidant systems.^25, 29, 30^ Thus, modulating the redox homeostasis represents a promising target in gastric cancer treatment.

It's reported that Cu-I exerts antitumorigenic activity by a ROS-mediated mechanism without inhibiting STAT3,^18, 23^ suggesting targeting the redox homeostasis may be an alternative mechanism of action for Cu-I in killing the cancer cells. In the present study, we show that Cu-I markedly inhibits gastric cancer cell growth by inducing G2/M phase cell cycle arrest and apoptosis at low nanomolar concentrations. The mechanistic study revealed that the effect of Cu-I is STAT3-independent. Our data further indicate that Cu-I elicits tumor-suppressive activity via a relative decrease in GSH content and, to a lesser extent, an increase of the GSSG content, which subsequently leads to a pro-oxidizing shift in the GSH/GSSG ratio. Our findings, however, disagree with Zhang *et al.*^18^ who suggested that ROS generation has an essential role in Cu-I-induced autophagy and cell death in HeLa cells. This apparent discrepancy may be explained by the fact that the basal levels of ROS in AGS and HGC-27 cells were >20 times and 10 times higher than that in HeLa cells, respectively (data not shown). We found a 1.3- or 1.4-fold increase of ROS accumulation in AGS or HGC-27 cells followed by Cu-I treatment for 6 h. Given the high basal ROS levels and impaired antioxidant capacity, such a marginal increase of ROS accumulation would also efficiently kill the gastric cancer cells. NRF2 and its downstream targets such as GCLM and G6PD are reported to be the most important regulator of GSH production.^10^ Our study, to the best of our knowledge, revealed for the first time that Cu-I could effectively inhibit the expression of NRF2 and its downstream targets GCLM and G6PD. The role of NRF2 in tumorigenesis has been identified in a broad spectrum of tumor types, such as ovarian, lung, breast, skin, and esophageal cancer.^31^ Some natural compounds, such as alkaloid trigonelline, which renders pancreatic cancer cells more susceptible to apoptosis, have been identified as NRF2 inhibitors.^32^ Our data suggest that Cu-I might be a novel inhibitor of the NRF2 pathway. The underlying mechanism by which Cu-I suppresses the NRF2 pathway in gastric cancer cells, however, remains unclear and will be investigated in our continued work.

GADD45*α* is a well-known p53-responsive stress protein to repair DNA damage, induce G2/M cell cycle arrest and apoptosis in various cancer cells.^14, 33^ However, the role of GADD45*α* in gastric cancer cells remains poorly understood. Our study demonstrates for the first time that Cu-I induced GADD45*α* through a p53-independent manner and subsequently led to G2/M cell cycle arrest and apoptosis in gastric cancer cells. GADD45*α* mRNA is induced in HGC-27 cells instead of AGS after Cu-I treatment, suggesting GADD45*α* is induced by Cu-I at multiple levels, either transcriptional or translational depending on the genetic context of cells. It has been proposed that Cu-I induced a considerable activation of JNK, p38 MAPK, and extracellular signal-regulated kinase after treatment in HeLa cells.^18^ Our study consistently revealed that Cu-I activated JNK and p38 MAPK in gastric cancer cells. Furthermore, our data collectively suggest the Cu-I induced GADD45*α* and JNK/p38 MAPK signaling form a positive feedback loop and can be reciprocally regulated by each other. Finally, and most importantly, Cu-I-induced GADD45*α* and JNK/p38 MAPK signaling were nearly completely eliminated when the cells were pretreated with NAC. Thus, our data strongly support a role for the decreased GSH/GSSG ratio by Cu-I in modulating this sophisticated loop (Figure 6f).

In the present study, we show for the first time that Cu-I has profound antigastric cancer activity both *in vitro* and *in vivo*. Instead of inhibiting the STAT3 pathway, Cu-I at low nanomolar concentrations induces cell cycle arrest and apoptosis in gastric cancer cell lines by decreasing the GSH/GSSG ratio, increasing the expression of GADD45*α* independent of p53 gene status, and activating JNK/p38 MAPK signaling. Taken together, our study provides new insight into the molecular effects of Cu-I in cancer treatment. Meanwhile, as dysregulation of redox balance that develops in cancer cells in response to increased pro-oxidants (ROS generation) and/or depletion of antioxidants serves as one important therapeutic target for the rational design of new anticancer agents,^34, 35^ our study provides a rationale for the development of Cu-I as a novel therapeutic agent against human gastric cancer.

## Materials and Methods

### Reagents, siRNAs, plasmids, and antibodies

Cu-I (C4493), *N*-acetyl-l-cysteine (NAC, A7250), DCF-DA (D6883), and l-Glutathione reduced (GSH, G4251) were purchased from Sigma-Aldrich (St. Louis, MO, USA). SP600125 (S1460), Z-VAD-FMK (S7023), and SB203580 (S1076) were purchased from Selleck Chemicals (Houston, TX, USA). DNA Damage Detection Kit was purchased from KeyGEN (KGA240). MitoSOX (M36008), SiRNAs target STAT3, and GADD45*α* as well as a negative control siRNA (sequences are detailed in Supplementary Table 1) were purchased from Invitrogen (Carlsbad, CA, USA). P53-responsive element luciferase reporter (pp53-TA-luc, D2223) and the negative control reporter pGL6-TA (D2105) were purchased from Beyotime Biotechnology (Jiangsu, China). Anti-cyclin B1 (4138), anti-cdc2 (9116), anti-cdk7 (2916), anti-caspase-3 (9662), anti-cleaved caspase-3 (9664), anti-caspase-8 (9746), anti-caspase-9 (9502), anti-JNK (9258), anti- phospho-(Thr183/Tyr185)-JNK (4668), anti-p38 (8690), anti-phospho-(Thr180/Tyr182)-p38 (4511), anti-STAT3 (9132), anti-phospho-(Tyr705)-STAT3 (9145), anti- phospho-(Ser15)-p53 (9286), and anti-phospho-(Ser139)-Histone H2AX (9718) antibodies were from Cell Signaling Technology (Beverly, MA, USA). Anti-GCLM (ab124827) and anti- Glucose 6 Phosphate Dehydrogenase (ab133525) were from Abcam (Cambridge, UK). Anti-phospho-(Tyr727)-STAT3 (P40763) antibody was from Bioworld Technology (St. Louis Park, MN, USA). Anti-PARP (sc-7150), anti-p53 (sc-6243), anti-p21 (sc- 397), anti-puma (sc-28226), anti-GADD45*α* (sc-797), and anti-NRF2 (sc-722) antibody were from Santa Cruz Technology (Santa Cruz, CA, USA). Anti-*β*-actin (A5441) antibody was from Sigma-Aldrich.

### Cell culture

The human normal gastric epithelial cell line GES-1, and gastric cancer cell lines AGS (p53-wild type) and HGC-27 (p53-mutated) were purchased from the cell bank of Chinese Academy of Sciences, and were authenticated by China Center for Type Culture Collection (CCTCC). All cell lines were cultured in RPMI-1640 medium supplemented with 10% FBS in a humidified incubator at 37 °C with 5% CO_2_.

### Cell viability assay

Cell viability was detected by CCK-8 assay. Cells were seeded into 96-well plates at 5 × 10^3^ cells/well and cultured overnight at 37 °C. After treatment with 0.1% DMSO as control or Cu-I at varying concentrations for indicated times, 10 *µ*l CCK-8 solutions were added to each well of the plate. Plates were incubated at 37 °C for 1 h, and then the absorbance at 450 nm was measured. All experiments were carried out in triplicate and repeated three times independently. The IC_50_ represented the drug concentrations leading to 50% cell growth inhibition and was calculated as described elsewhere.^12^

### Cell cycle assay

About 3 × 10^5^ cells/well were seeded in a six-well plate overnight at 37 °C, and then treated with indicated concentrations of Cu-I for 12 h. After harvesting the cells, cells were immediately stained according to the instructions of Cycle TEST DNA Reagent Kit (340242, BD Biosciences, San Jose, CA, USA). The phase of cell cycle was analyzed by flow cytometry (BD Biosciences, Aria II). Data were analyzed with BD FACSDiva Software. All experiments were done in triplicate and repeated three times independently.

### Apoptosis assay

Apoptosis induced by Cu-I was assessed by flow cytometry using FITC Annexin V Apoptosis Detection Kit (556547, BD Biosciences). Similarly, cells were treated as done in the cell cycle assay. All operations were performed as previously described.^12, 36^ All experiments were done in triplicate and repeated three times independently.

### Colony-formation assay

Colony-formation assay was performed as previously described.^37^ In brief, ~1 × 10^3^ cells/well were seeded into six-well plates overnight and then incubated with indicated concentrations of Cu-I. After 14 days, the colonies were fixed with methanol and stained with crystal violet. Crystal violet stained colonies were photographed. All experiments were done in triplicate and repeated three times independently.

### Western blotting

Cell lysates were collected as previously described.^38^ Thirty-microgram lysates were separated on 6–12% sodium dodecyl sulfate-polyacrylamide gels and transferred to PVDF membranes (Millipore, Bedford, MA, USA). TBST containing with 5% nonfat milk or bovine serum albumin was used to block nonspecific binding for 2 h at room temperature. Then membranes were incubated with primary antibodies according to the instructions overnight at 44 °C, followed by appropriate HRP-conjugated secondary antibodies (1:5000 dilutions). Signals generated by enhanced chemiluminescence (Millipore) were recorded with a CCD camera (CLINX, Shanghai, USA). Data are representative of at least three independent experiments.

### RNA Extraction, reverse transcription, and Real-time PCR

Total RNA was extracted and reversely transcribed to cDNA as described previously.^38^ Real-time quantitive PCR assays was conducted using the SYBR Premix Ex TaqTM II kit (TaKaRa, Shuzo Co., Ltd., Kyoto, Japan). The real-time PCR experiments were performed with ABI PRISM 7500 Fast Real-Time PCR System. Primers used in real-time PCR experiments were shown in Supplementary Table 2.

### Transfection of AGS cell with siRNA

Transfection was carried out using Lipofectamine RNAiMax Reagent (Invitrogen) as described elsewhere (reverse transfection method).^39^ In brief, 50 pmol siRNA and 0.5 ml Opti-MEM I Medium (Invitrogen) without serum was mixed in each well of six-well plate. Then 7.5 *μ*l of Lipofectamine RNAiMAX reagent was added and gently mixed. After incubation for 20 min at room temperature, 2 ml of cell suspension including 3 × 10^5^ cells in complete growth medium without antibiotics was added into each well. This gives a final siRNA concentration of 20 nM.

### DNA transfection and luciferase reporter assay

DNA transfection and luciferase reporter assay were done as previously described.^37^ Cells were plated to 24-well plates 24 h prior to transfection experiments. Transfection experiment was carried out by adding 380 ng of reporter plasmid along with a pRL-TK reporter plasmid (20 ng) as a control for transfection efficiency. All assays were performed by using a Dual Luciferase Reporter Assay System (Promega, Madison, WI, USA).

### ROS and glutathione assay

For analyses of cellular ROS, 5 × 10^3^ cells or 3 × 10^5^ cells/well were seeded in a 96-well plate or six-well plate, then treated by Cu-I (0–200 nM) as indicated time, respectively. The redox-sensitive probe DCF-DA (5 *µ*M) was incubated for 30 min after treatment. Fluorescence intensity was measured immediately by fluorescence microplate assay in 96-well plates as described^40^ or by flow cytometry analysis in six-well plates-treated cells. For highly selective detection of superoxide in the mitochondria of cells, Cu-I-treated cells were incubated with 5 *µ*M MitoSOX for 30 min in HBSS, washed twice with PBS and analyzed with flow cytometry (BD Biosciences, Aria II).^18^ For analyses of tissue ROS, tumors were frozen in optimum cutting temperature compound, cryosectioned, and incubated with 5 *µ*M DCF-labeled redox-sensitive probe for 90 min at 37 °C, and photographed with the same exposure time as described.^41^ The levels of GSH and GSSG were measured in cell or tumor lysates according to the instructions of GSH and GSSG Assay Kit (Beyotime Biotechnology, S0053). All experiments were done in triplicate and repeated three times independently.

### Single-cell gel electrophoresis (SCGE comet assay)

After 12 h or 24 h treatment, AGS cells were harvested and suspended in PBS for the SCGE comet assay, which was performed to determine the degree of DNA damage. All operations were performed as described in our previous study.^42^

### Tumor xenograft model in nude mice

All animal procedures and care were approved by the Institutional Animal Care and Use Committee of Nanjing Drum Tower Hospital, Medical School of Nanjing University. HGC-27 cells (~3 × 10^6^ cells/mice) were resuspended in 100 *μ*l serum-free medium and gently mixed with the same volume of ice-cold Matrigel, and were injected subcutaneously into the left flank of 4- to 6-week-old female BALB/c nu/nu nude mice (*n*=10). Tumor size was measured as previously described.^36^ After 5 days when the tumor size reached ~100 mm^3^, mice were randomly assigned into Cu-I treatment group (receiving 1 mg/kg of Cu-I in 5% DMSO in PBS by intraperitoneal injection daily, *n*=5) and control group (receiving vehicle containing the same percentage of DMSO in PBS, *n*=5). Tumor volume and animal weight were measured every 4 days. Mice were killed when control group tumors reached ~1000 mm^3^. Tumors and organs including liver, spleen, lung, and kidney were harvested. All excised tumors were weighed, frozen in liquid nitrogen or fixed in 10% neutral formalin and embedded in paraffin for further immunohistochemical or immunofluorescence studies.

### Statistics

All experiments were repeated at least three times in triplicate. Each data is represented as mean±S.D. GraphPad Prism software v.6.01 with one-way analysis of variance (ANOVA) with Dunnett's multiple comparisons test and two-way ANOVA with Tukey's multiple comparisons test was used in corresponding statistical evaluations (*P<0.05; **P<0.01).

## Abbreviations

CDC2: cell division cycle gene 2
DCF-DA: 2',7'-dichlorofluorescein diacetate
GADD45*α*: growth arrest and DNA-damage-inducible protein 45*α*
GCLM: glutamate–cysteine ligase complex modifier subunit
GSH: reduced glutathione
GSSG: oxidized glutathione
G6PD: glucose-6-phosphate dehydrogenase
JNK: c-Jun NH2-terminal kinase
MAPK: mitogen-activated protein kinase
NAC: *N*-acetyl-l-cysteine
NRF2: nuclear factor erythroid 2-related factor 2
PARP: poly(ADP-ribose) polymerase
ROS: reactive oxygen species
STAT3: signal transducer and activator of transcription 3
