# Supplementary figures and images for: Low nanomolar concentrations of Cucurbitacin-I induces G2/M phase arrest and apoptosis by perturbing redox homeostasis in gastric cancer cells in vitro and in vivo
Source: Cell Death Dis. 2016 Feb 18;7(2):e2106–. doi: 10.1038/cddis.2016.13 (PMC5399186; doi:10.1038/cddis.2016.13)

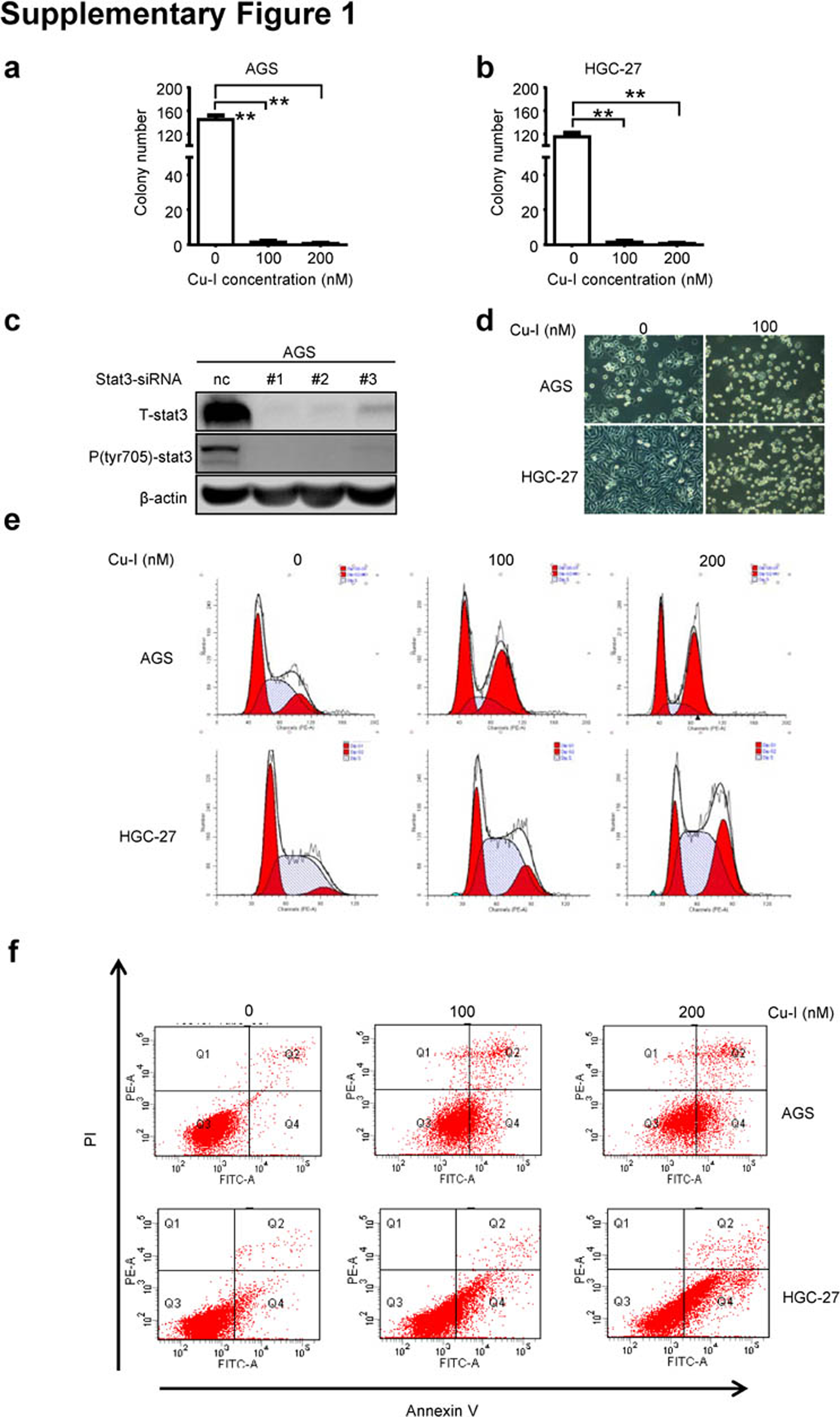

Supplement: Supplementary Figure 1 [file cddis201613x1.tif]

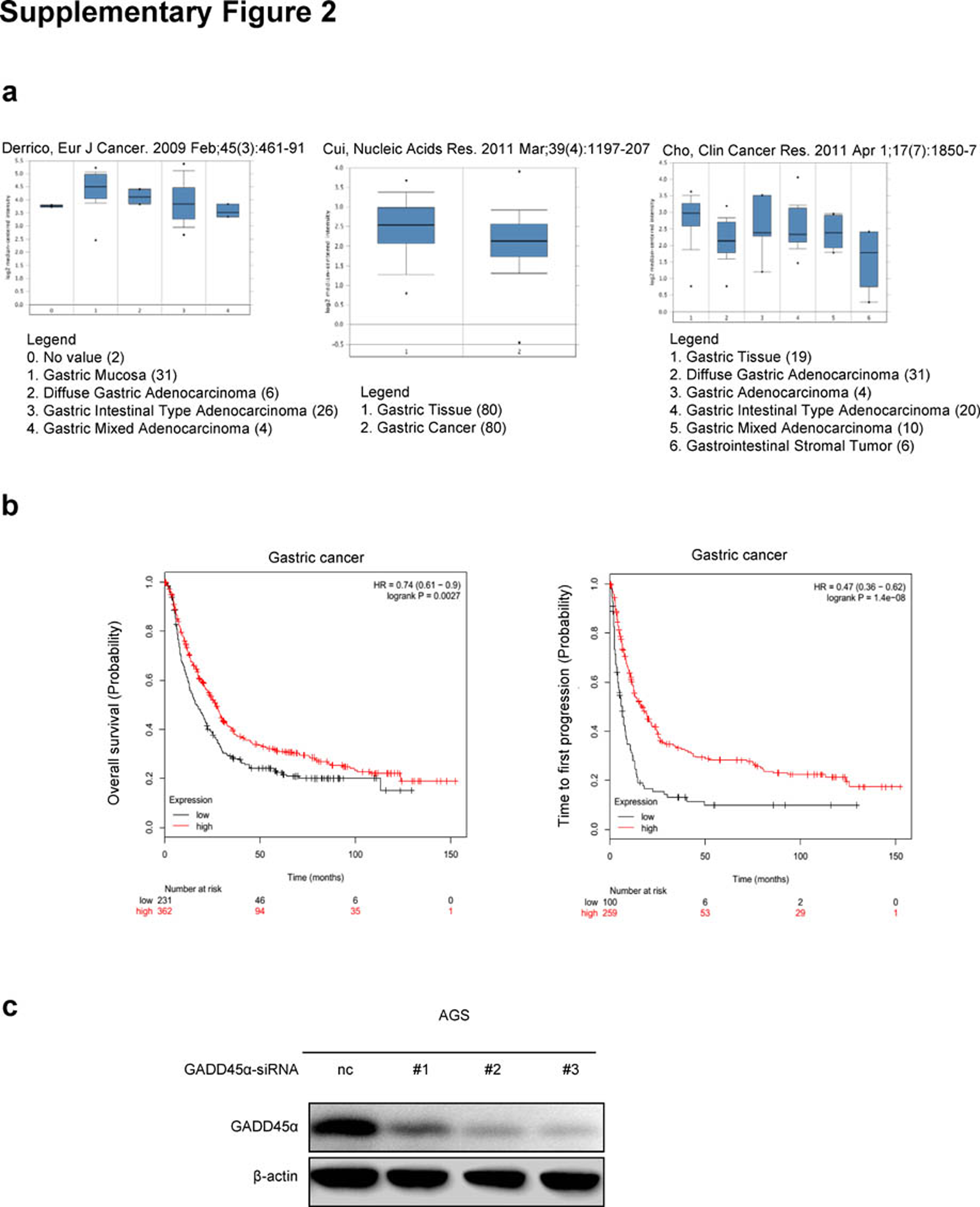

Supplement: Supplementary Figure 2 [file cddis201613x2.tif]

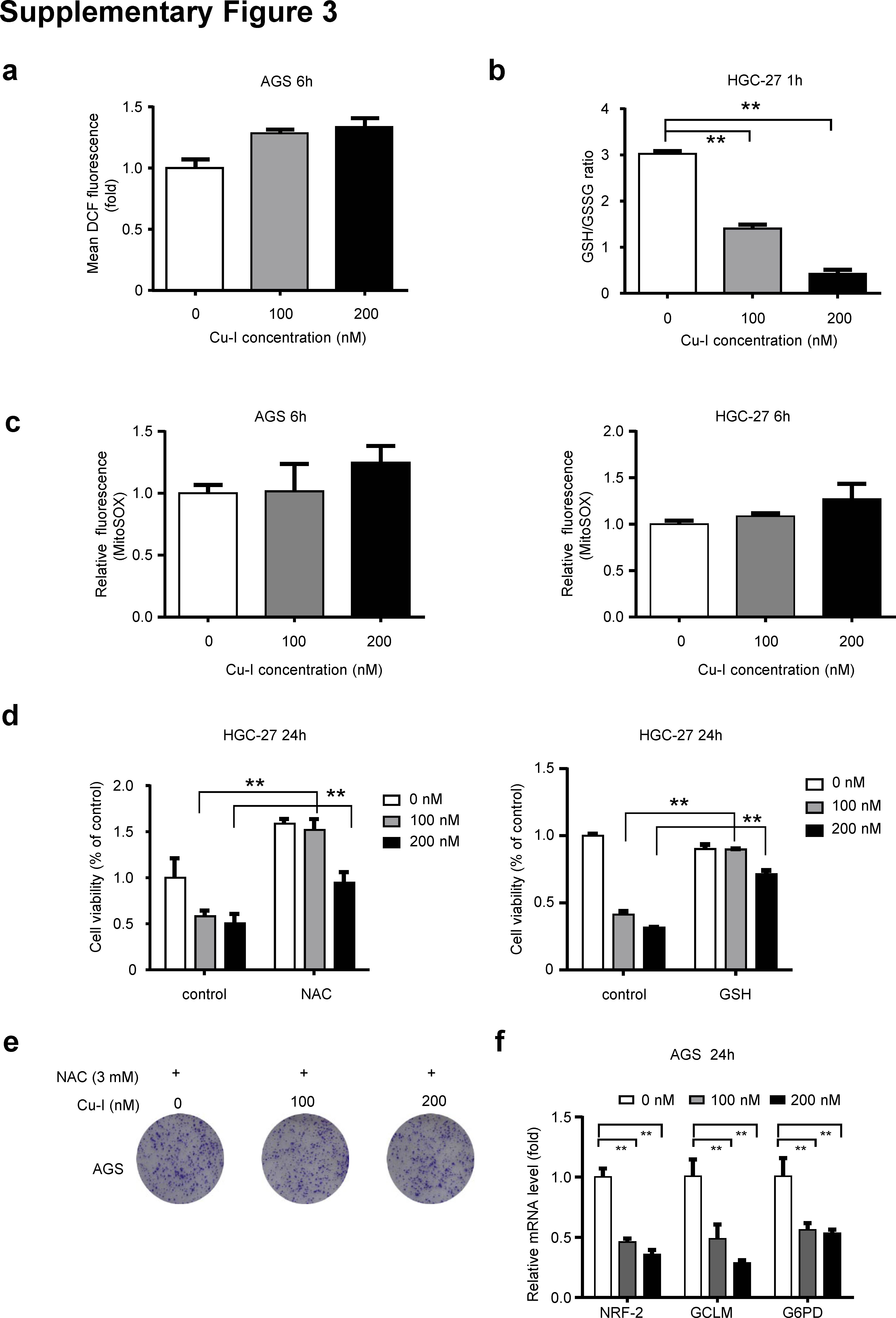

Supplement: Supplementary Figure 3 [file cddis201613x3.tif]

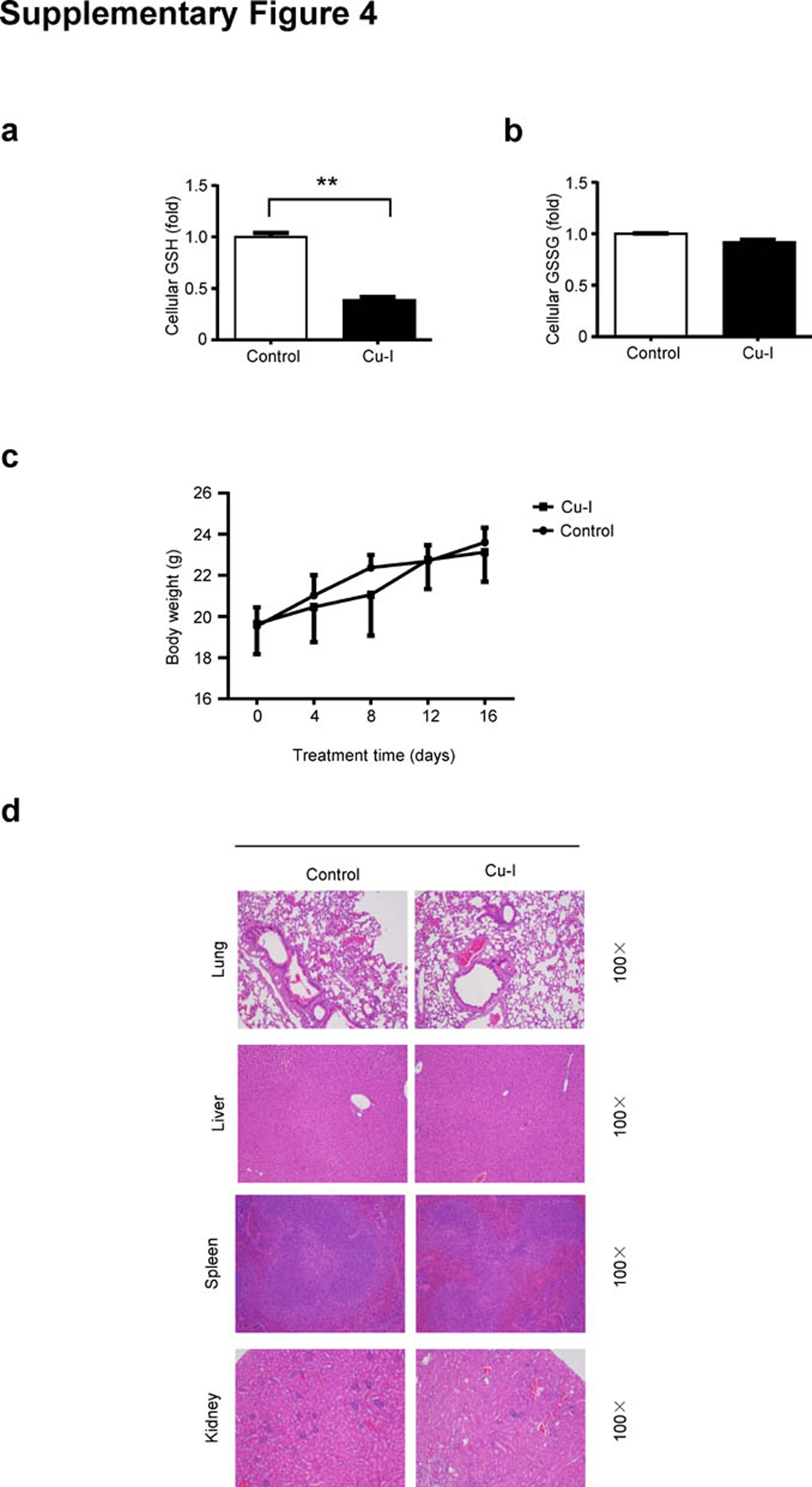

Supplement: Supplementary Figure 4 [file cddis201613x4.tif]

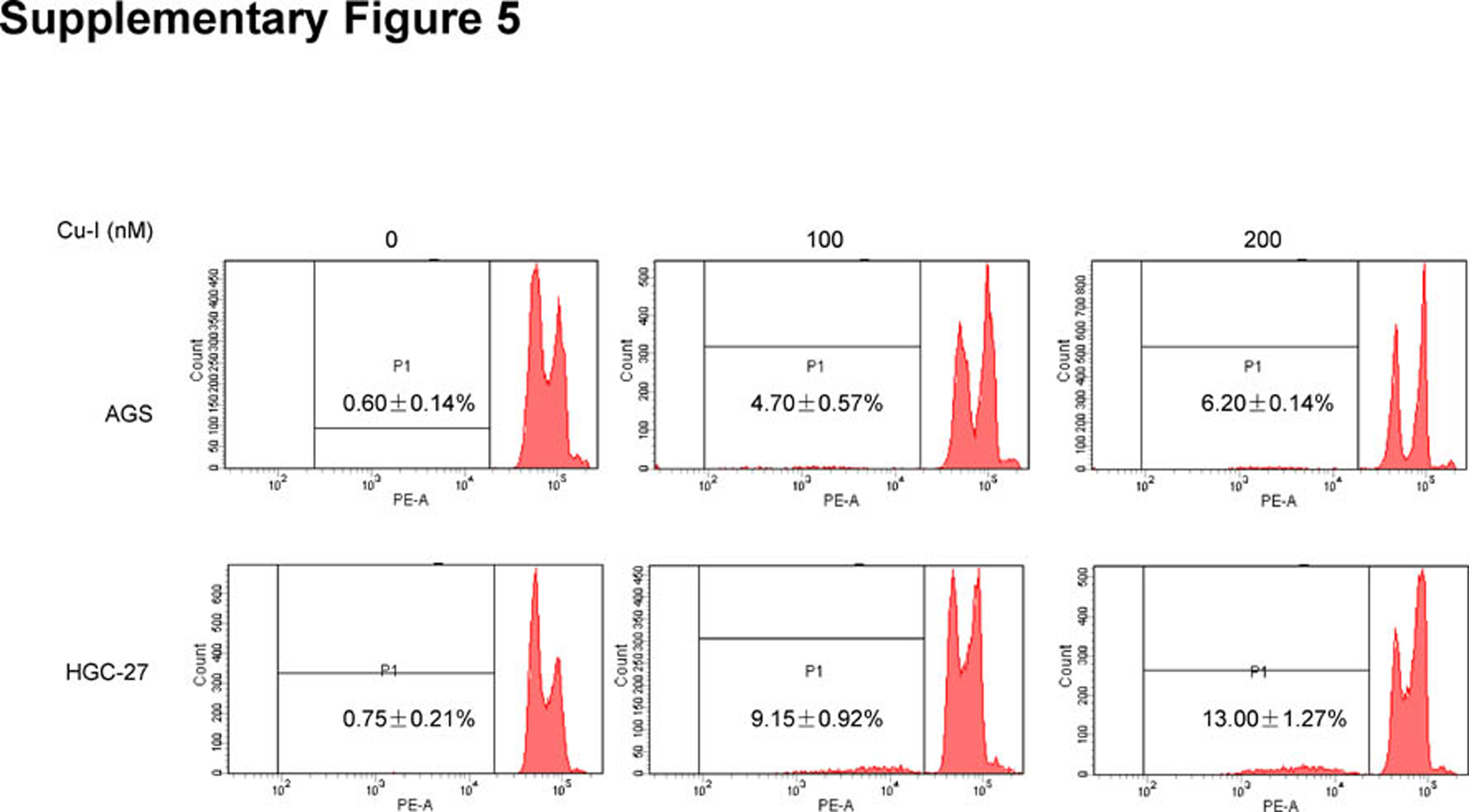

Supplement: Supplementary Figure 5 [file cddis201613x5.tif]
