# Supplementary material for: Low nanomolar concentrations of Cucurbitacin-I induces G2/M phase arrest and apoptosis by perturbing redox homeostasis in gastric cancer cells in vitro and in vivo
Source: Cell Death Dis. 2016 Feb 18;7(2):e2106–. doi: 10.1038/cddis.2016.13 (PMC5399186; doi:10.1038/cddis.2016.13)
Supplement: Supplementary Table 1 [file cddis201613x6.doc]

Supplementary table 1 The sequences of STAT3 and GADD45α siRNA.

| Gene | Sequence |
| --- | --- |
| STAT3 |  |
| si#1 | 5’-GCCAATTGTGATGCTTCCCTGATTG-3’ |
| si#2 | 5’-TGGCCCAATGGAATCAGCTACAGCA-3’ |
| si#3 | 5’-GATAACGTCATTAGCAGAATCTCAA-3’ |
| GADD45α |  |
| si#1 | 5’-GCCGAAAGGGTTAATCATATT-3’ |
| si#2 | 5’-GCTGAGTGAGTTCAACTACAT-3’ |
| si#3 | 5’-GAAGACCGAAAGGATGGATAA-3’ |
