# Supplementary material for: Low nanomolar concentrations of Cucurbitacin-I induces G2/M phase arrest and apoptosis by perturbing redox homeostasis in gastric cancer cells in vitro and in vivo
Source: Cell Death Dis. 2016 Feb 18;7(2):e2106–. doi: 10.1038/cddis.2016.13 (PMC5399186; doi:10.1038/cddis.2016.13)
Supplement: Supplementary Table 2 [file cddis201613x7.doc]

Supplementary table 2 PCR primers used in this study.

| Gene | Primer sequences (5′–3′) |
| --- | --- |
| β-actin-FW | CTGG CACCACACCTTCTACAATG |
| β-actin-RV | AATGTCACGCACGAT TTCCCGC |
| GADD45α-FW | ATGGATAAGGTGGGGGATGC |
| GADD45α-RV | ACGTTATCGGGGTCGACGTT |
| NRF2-FW | GGCCCATTGATGTTTCTGAT |
| NRF2-RV | TTAGTGAAATGCCGGAGTCA |
| GCLM-FW  GCLM-RV  G6PD-FW  G6PD-RV | GCCACCAGATTTGACTGCCTTT  CAGGGATGCTTTCTTGAAGAGCTT  TGAGCCAGATAGGCTGGAAC  GGTAGTGGTCGATGCGGTAG |
