# Supplementary material for: Low nanomolar concentrations of Cucurbitacin-I induces G2/M phase arrest and apoptosis by perturbing redox homeostasis in gastric cancer cells in vitro and in vivo
Source: Cell Death Dis. 2016 Feb 18;7(2):e2106–. doi: 10.1038/cddis.2016.13 (PMC5399186; doi:10.1038/cddis.2016.13)
Supplement: Supplementary Figure Legend [file cddis201613x8.doc]

Supplementary Figure 1 (a and b) Quantitative data shown as mean ± SD of the colony formation assay of Figure 1c. (c) Lysates of AGS cells transfected with either negative control siRNA or STAT3-siRNAs for 48 h was evaluated by western blotting with indicated antibodies. (d) Representative images of cell morphological changes of AGS and HGC-27 cells after about 1 h treatment with 100 nM of Cu-I. (e) Representative images of cell cycle analysis in Cu-I-treated AGS and HGC-27 cells. (f) Representative images of Annexin V/PI double staining in Cu-I-treated AGS and HGC-27 cells.

Supplementary Figure 2 The web-based human cancer databases analysis of GADD45α mRNA expression in human gastric cancer and relationship associated with survival and prognosis. (a) Oncomine dataset (<https://www.oncomine.org/resource/main.html>) were used to compare the expression of GADD45α mRNA in normal gastric tissue with that in gastric cancer from studies reported by Derrico et al., Cui et al., and Cho et al. (b) Kaplan–Meier plot showing overall survival and time to first progression of gastric cancer patients stratified by high or low GADD45α mRNA expression. These data are from 593 gastric tumor samples using publicly available datasets (<http://kmplot.com/analysis/index.php?p=service&cancer=gastric>). (c) SiRNAs targeting GADD45α mRNA to knockdown its endogenous expression were validated by Western blotting.

Supplementary Figure 3 (a) AGS cells were treated by Cu-I (0-200 nM) for 6 h, fluorescence intensity was measured as described in Figure 5d. (b) The ratio of GSH/GSSG was calculated in HGC-27 cells after Cu-I treatment for 1 h. ** *p* <0.01. (c) Cu-I-treated AGS and HGC-27 cells were stained with MitoSOX (5 uM) for 30 min and mitochondrial superoxide generation was determined using a flow cytometry. (d) HGC-27 cells were treated as described in Figure 5f for 24 h. The viability of cells was assayed by CCK-8. ** *p* <0.01, compared with Cu-I alone-treated group. (e) AGS cells treated as described in Figure 5f were grown for 14 days and stained with 0.5% crystal violet to visualize colonies. (f) The mRNA levels of NRF2, GCLM and G6PD in AGS cells treated by Cu-I for 24 h were assayed by real-time PCR.

Supplementary Figure 4 (a and b) Tumor lysates analysis of levels of GSH and GSSG in each group. (c) The body weight of mice in each group was measured every 4 days to monitor the side-effects of Cu-I. (d) After the mice were sacrificed, the vital organs (lung, liver, spleen and kidney) were collected for histopathological analysis.

Supplementary Figure 5 Re-analysis of cell cycle data to show the portion of cells within sub-G1.
